# Increased skin autofluorescence of children and adolescents with type 1 diabetes despite a well-controlled HbA1c: results from a cohort study

**DOI:** 10.1186/s12902-016-0129-3

**Published:** 2016-09-09

**Authors:** Josine C. van der Heyden, Erwin Birnie, Dick Mul, Sarah Bovenberg, Henk J. Veeze, Henk-Jan Aanstoot

**Affiliations:** 1 Diabeter, Center for Pediatric and Adolescent Diabetes Care and Research, Blaak 6, 3011 TA Rotterdam, Netherlands; 2 Department of Pediatric Endocrinology, Sophia Children’s Hospital, Erasmus MC, University Medical Centre, Wytemaweg 80, 3015 CN Rotterdam, Netherlands; 3 Department of Pediatrics, Sint Franciscus Gasthuis, Kleiweg 500, 3045 PM Rotterdam, Netherlands; 4 Department of Genetics, University Medical Center Groningen, University of Groningen, Hanzeplein 1, 9713 GZ Groningen, Netherlands

**Keywords:** Pediatrics, Type 1 diabetes, Skin autofluorescence, Skin advanced glycation end products, Diabetic complications

## Abstract

**Background:**

Early identification of children and adolescents with type 1 diabetes at high risk for development of complications is important, as early intervention may prevent further deterioration. Here we investigate the applicability of assessing skin advanced glycation end products (sAGEs) by skin autofluorescence (SAF) as a potential surrogate risk marker.

**Methods:**

This study included a cross-sectional analysis of SAF in 77 patients with type 1 diabetes mellitus and 118 healthy controls across age categories (11–12, 13–14, 15–16, and 17–19 years old). In patients, the impact of current and historical glycated hemoglobin (HbA1c) values, age, and duration of diabetes on SAF was studied in a retrospective cohort study and analyzed with multivariable analyses.

**Results:**

SAF was significantly and similarly higher in patients when compared with controls across all age categories (*P* ≤0.009). For patients, age, duration of diabetes, and current and historical HbA1c were associated with SAF in univariate analysis. Multivariate analysis showed no association between HbA1c and SAF. A subgroup of patients with a HbA1c-within-target (≤7.5 %/59 mmol/mol) were observed to have high SAF.

**Conclusion:**

Children and adolescents with type 1 diabetes show higher SAF than controls. The presumed correlation of high HbA1c with high SAF does not exist in all patients. Thus, use of this non-invasive measure may provide a surrogate marker for diabetic complications, additional to HbA1c.

## Background

Reactive oxygen species and non-enzymatic reactions between sugars and amino groups of proteins (‘Maillard reaction’) are involved in the formation of advanced glycation end products (AGEs) [1]. AGEs cause oxidative stress-related tissue damage [1], which plays an important role in microvascular and macrovascular complications in diabetes [2].

As skin collagen has a half-life of 10–15 years [3], skin AGEs (sAGEs) represent long-term glycemia. Accumulation of sAGEs can be assessed easily and non-invasively by measuring skin autofluorescence (SAF) [4] or skin intrinsic fluorescence (SIF) [5, 6]. sAGEs have been proposed as a surrogate measure for risk assessment additional to HbA1c in patients with diabetes [7]. In adults, increased levels of sAGEs were found in patients who developed complications [7–9]. As early intervention may prevent damage later in the disease course of diabetes [10], identification of young patients at high risk for micro- and macrovascular complications is of paramount importance [11]. SAF/SIF may be an effective measurement to identify this disadvantaged group. Previous studies assessed SAF/SIF in a rather heterogeneous group of children and adolescents with type 1 diabetes [12, 13], but some lacked a proper control group [14, 15]. Felipe et al. [5] found SIFto be weakly associated with mean HbA1c of the preceding period and with diabetes duration.

Here we investigate if SAF reflects glycemic control expressed by HbA1c in a homogeneous study population. SAF in Dutch Caucasian children and adolescents with type 1 diabetes was compared with SAF in healthy Caucasian controls. In patients, associations of SAF with age, diabetes duration, gender, and current and historical (past) HbA1c as a reflection of long-term glycemic control were determined. We hypothesize that SAF will be higher in patients compared with controls and that it will associate with the variables age, historical HbA1c, and diabetes duration.

## Methods

### Study design and population

The study design was a retrospective cohort study of patients with type 1 diabetes and healthy controls. Patients aged 11–19 years with type 1 diabetes ≥3 months were recruited between April 2010 and January 2013 while visiting the outpatient clinic of Diabeter, a certified center of reference for diabetes care in Rotterdam, Netherlands. Diabeter provides comprehensive and advanced management for children and adolescents with type 1 diabetes. Patients were only included if they were Caucasian and if measurements of SAF and HbA1c were performed on the same day. Patients with inadequately controlled celiac disease, hypothyroidism, and those using lipid-lowering therapy were excluded.

The healthy control group was recruited from a secondary school in Rotterdam in October and November 2011. Controls with missing data, non-Caucasian ethnicity, and concomitant diseases other than attention deficit/hyperactivity disorder were excluded. Study participants and parents of minors provided signed informed consent. The study was approved by the Medical Ethical Board of the Erasmus Medical Center, Rotterdam, Netherlands, and performed in accordance with the Declaration of Helsinki.

### Anthropometric and laboratory data

In controls, information on gender, ethnic background, and concomitant diseases was collected. Anthropometric and laboratory data were not obtained for this group. In patients, information on duration of diabetes, gender, body mass index (BMI), blood pressure, current HbA1c, and HbA1c values in the past (called ‘historical HbA1c’ from here on) was retrieved from electronic patient charts. Information on BMI and blood pressure was included if measurements were performed within a time interval of 1 month around the SAF measurement. BMI was converted to standard deviation scores (SDS): a high BMI was defined as a BMI ≥+2 SDS. A normal BMI was defined as a BMI <+2 SDS [16–18]. Systolic blood pressure (SBP) and diastolic blood pressure (DBP) were converted to percentiles [19]. HbA1c was measured at every clinic visit by immunochemical assay (Vantage System, Siemens Medical Solutions Diagnostics, Tarrytown, NY) with intra- and inter-assay coefficients of variation of <3.7 % and <4.3 %, respectively. Current HbA1c was defined as the HbA1c measured on the same day as SAF. Historical HbA1c was defined as the median intrapersonal HbA1c value of multiple HbA1c data included from the first clinic visit onwards. Historical HbA1c was only determined if the first HbA1c measurement was done ≥3 months after the diagnosis of type 1 diabetes and if ≥3 HbA1c measurements were done in the period from the first measurement to the current HbA1c measurement (current HbA1c measurement not included). A current or historical HbA1c >7.5 %/59 mmol/mol was defined as ‘HbA1c-above-target’ whereas an HbA1c of ≤7.5 %/59 mmol/mol was defined as (relatively) ‘Hba1c-within-target’ [20].

### SAF measurements

Patient SAF measurements were performed at Diabeter. Measurements of controls were performed at their school. The volar side of the forearm was measured with the AGE Reader CU autofluorescence reader (Diagnoptics BV, Groningen, Netherlands), making sure that the site of measurement was clean. The autofluorescence reader illuminates a skin surface of approximately 1 cm^2^ with an excitation light source between 300 and 420 nm (peak excitation ∼ 350 nm) [21]. Three independent measurements were performed in approximately 30 s: the arm was repositioned between measurements. The mean of the three measurements was displayed by the SAF reader in arbitrary units. Previously, Sugisawa et al. [22] reported a coefficient of diurnal variation of 3.7 % and a coefficient of daily variance of 4.6 %.

### Statistical methods

Due to the lack of prior data on SAF measurements in patients and controls as well as absence of data on the relationship between HbA1c and SAF in diabetes patients, formal sample size could not be calculated at study onset. Instead, our aim was to include at least 100 students and at least 70 diabetics during the study period.

Normal distributions were expressed as mean with SD. Continuous variables with skewed distributions were expressed as median with interquartile range (IQR). Categorical variables were expressed as proportions and percentages. Differences in continuous variables between groups were tested with the Mann-Whitney U test. Differences in categorical variables between groups were tested by the chi-squared test or Fisher’s exact test. Correlations were tested with Pearson’s rho in case of normal distribution and Spearman’s rho in case of skewed distribution. Patients with type 1 diabetes and controls were stratified into multiple age categories (11–12, 13–14, 15–16, and 17–19 years). For each age category, patients were stratified according to HbA1c-above-target and Hba1c-within-target. For both patients with HbA1c-above-target and Hba1c-within-target, SAF was descriptively compared with SAF of controls.

Univariate and multiple linear regression analyses were performed. Univariate linear regression analyses were used to assess the impact of the following covariables on SAF: age; diabetes duration (≤4 years, 4 to <10 years, and ≥10 years); gender; current HbA1c (current value, and HbA1c-above-target/Hba1c-within-target); and historical HbA1c (historical value, and HbA1c-above-target/Hba1c-within-target). BMI was not included due to low variability in BMI values. SBP and DBP were not included because no associations with SAF were found. Multiple linear regression analyses were performed using two models. Model 1 assessed the impact of age and diabetes duration on SAF. In model 2, current and historical HbA1c were added to the model. A *P* value <0.05 was considered statistically significant.

## Results

### Study population

**Fig. 1:**
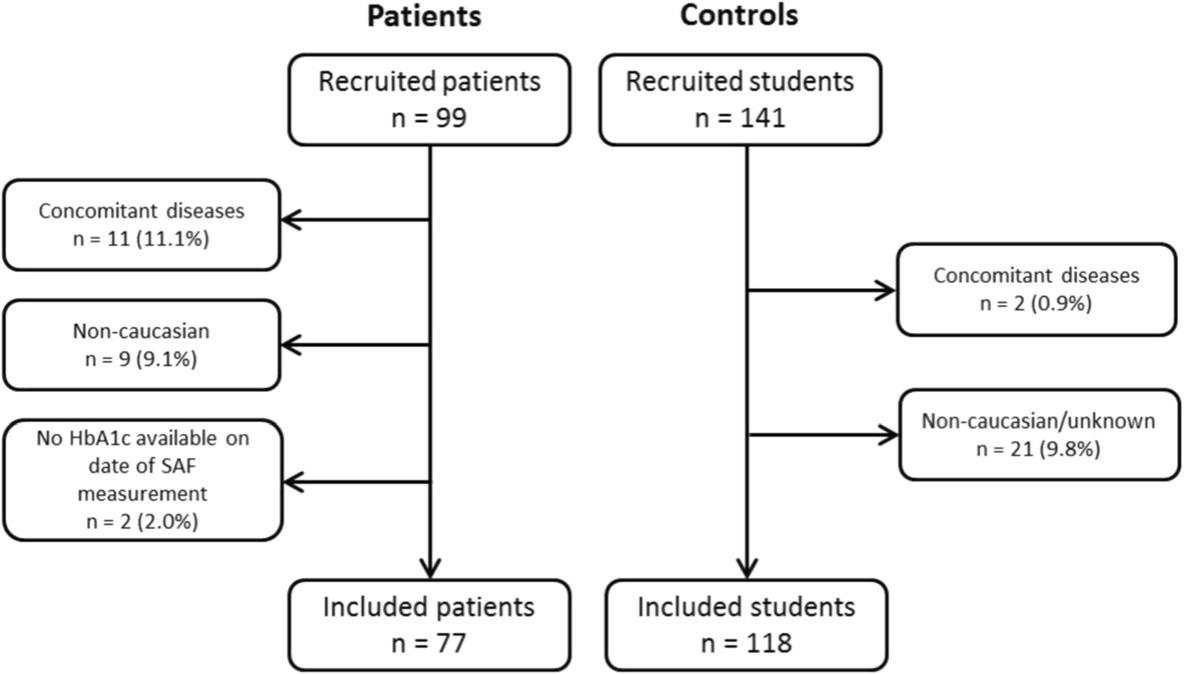
Study profile. *SAF* skin autofluorescence

A total of 99 patients with type 1 diabetes and 141 controls were recruited, of whom 77 patients and 118 healthy controls were included (Fig. 1). Baseline characteristics of patients and controls are presented in Table 1. The median age of patients with type 1 diabetes was higher than controls (*P* = 0.004). Females were significantly overrepresented in the control group (*P* = 0.042). Historical HbA1c was determined in 73 patients (median 26 HbA1c measurements; IQR 17–37; range 3–71) in a period of 4.12 years (IQR 2.43–6.07 years; range 0.75–13.88 years).

**Table 1:** Baseline characteristics of patients with type 1 diabetes and healthy controls

	Patients: *n* = 77	Controls: *n* = 118
Age, years	15.3 (13.6–17.0)	14.4 (13.1–15.4)
Gender: female, n (%)	39 (50.6 %)	77 (65.3)
Diabetes duration, years (IQR)	6.6 (3.5–9.5)	NA
Age at diabetes onset, years (IQR)	8.9 (5.7–11.4)	NA
SBP (percentile)	78 (49–91)^a^	NA
DBP (percentile)	37 (21–66)^a^	
BMI, n (%)		NA
≤ −2 SDS	-	
> −2 SDS and <0 SDS	16 (21.6)^b^	
≥0 SDS and <+2 SDS	55 (74.3)^b^	
≥ +2 SDS	3 (4.1)^b^	
HbA1c Current	8.46 (1.35)	NA
Median Intrapersonal Historical HbA1c, % (IQR)	8.0 (7.48–8.60)^c^	NA

^a^ *n* = 69

^b^ *n* = 74

^c^ *n* = 73

*DBP* diastolic blood pressure, *IQR* interquartile range, *SBP* systolic blood pressure, *SDS* standard deviations score

### SAF measurements in controls and patients

**Fig. 2:**
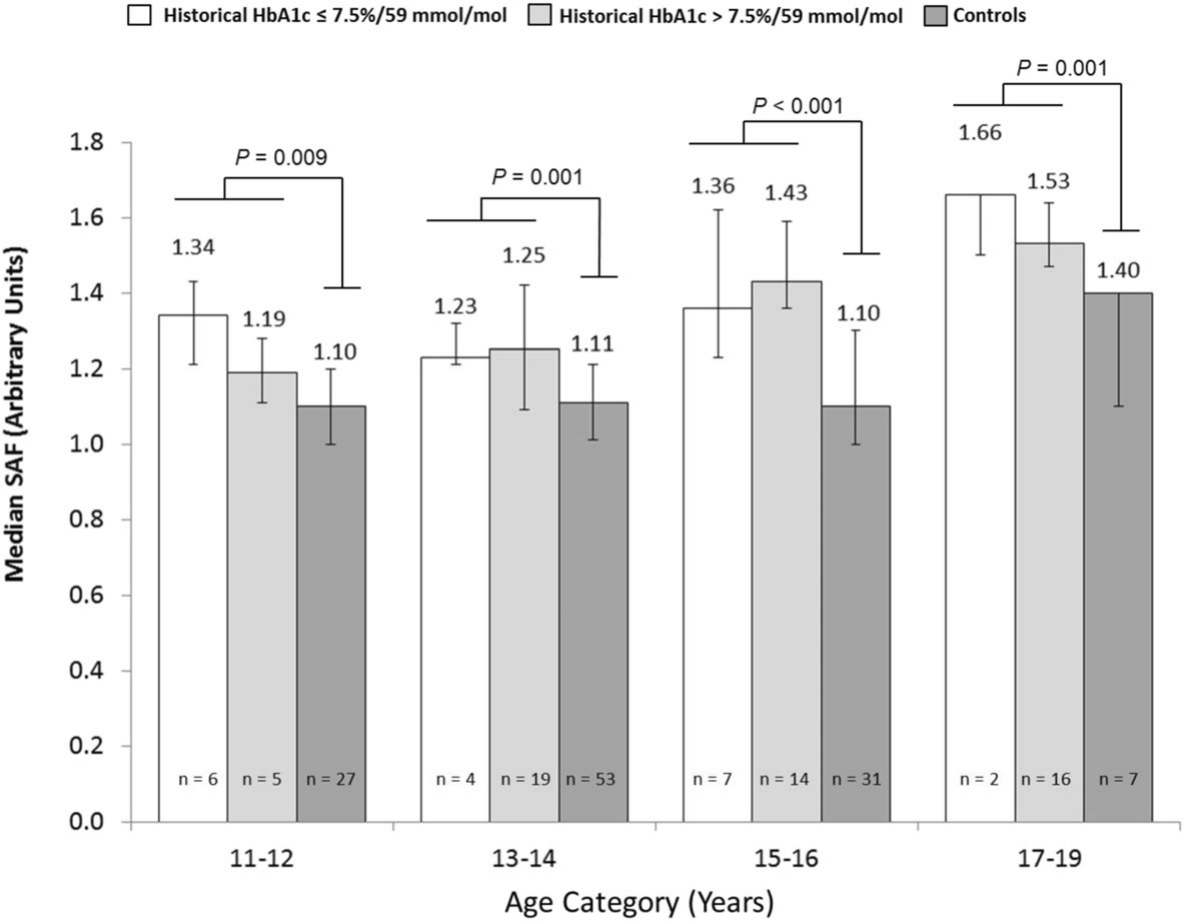
Age-specific median skin autofluorescence (SAF) in patients with a HbA1c-above-target (>7.5 %/59 mmol/mol) and a HbA1c-within-target (≤7.5 %/59 mmol/mol), compared with controls. *P*-values: comparison of patients vs controls per age category by Mann-Whitney U test. Error bars: interquartile range

Figure 2 shows SAF measurements per age category for the controls, and for the patients with historical HbA1c-above-target and Hba1c-within-target. Both the overall SAF (patients: median [IQR] SAF 1.40 [1.23–1.54]; controls: mean [SD] SAF 1.14 [0.14], *P* <0.001) and the median SAF in the different age categories were significantly higher in patients when compared to controls (Fig. 2). In the control group, median SAF increased from 1.10 [IQR 1.00–1.20] in the age category 11–12 years to 1.40 [IQR 1.10–1.40] in the age category 17–19 years. In the patient group, the same pattern was seen: in the age category 11–12 years, median SAF was 1.24 [IQR 1.19–1.40], and in the age category 17–19 years, median SAF was 1.53 [IQR 1.48–1.66] (Fig. 2).

### Impact of HbA1c on SAF in patients

Within the patient group, median SAF of patients with a current HbA1c-within-target (*n* = 13) was 1.32 [IQR 1.19–1.52] and of patients with a historical HbA1c-above-target (*n* = 64) 1.40 [IQR 1.22–1.54](*P =* 0.654). In the same group, median SAF of patients with a historical HbA1c-within-target (*n* = 19) was 1.36 [IQR 1.23–1.50] and of patients with a historical HbA1c-above-target (*n* = 54) 1.41 [1.23–1.54](*P =* 0.580). There were also no statistically significant differences in SAF between the HbA1c-above-target and HbA1c-within-target patients for the various age categories (Fig. 2).

**Fig. 3:**
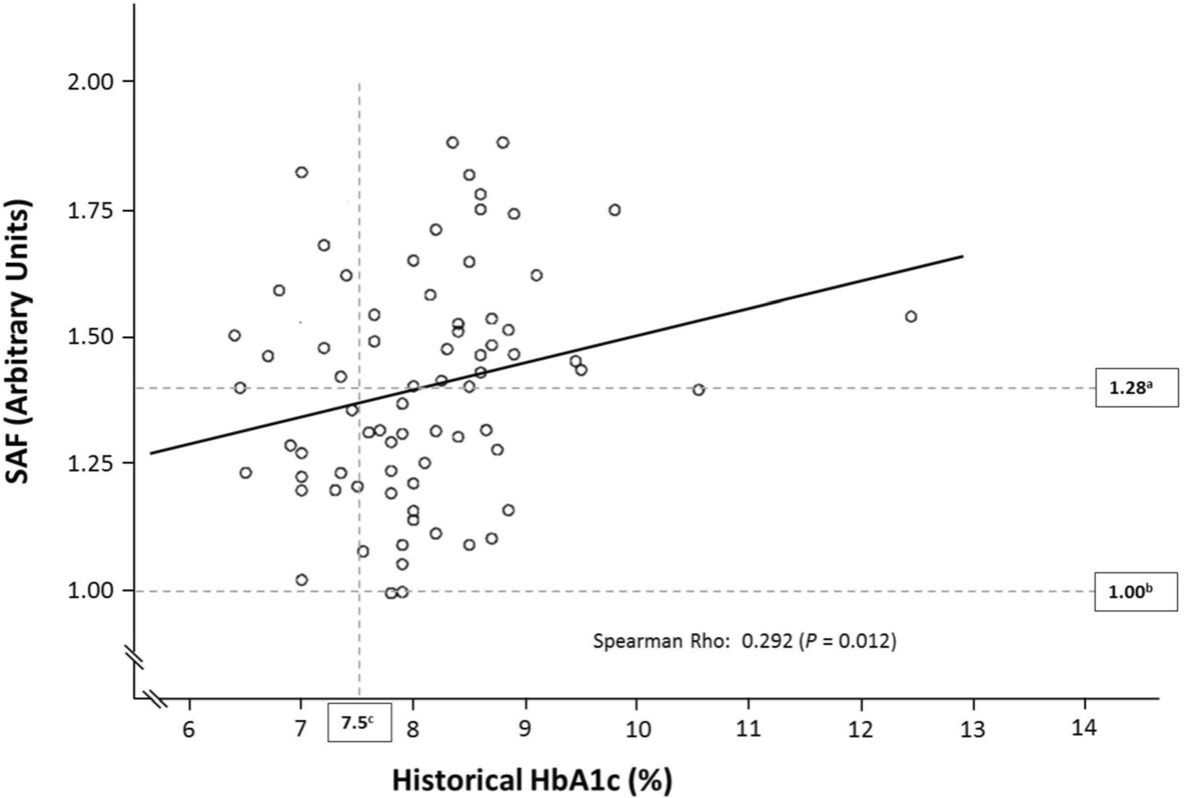
Correlation between historical HbA1c and skin autofluorescence (SAF) in children and adolescents with type 1 diabetes. ^a^mean SAF of controls + 1 SD. ^b^mean SAF of controls -1SD. ^c^HbA1c-within-target (≤7.5 %/59 mmol/mol) vs HbA1c-above-target (>7.5 %/59 mmol/mol)

Figure 3 shows the association between historical HbA1c and SAF. The Spearman correlation coefficient between historical HbA1c and SAF was 0.292 (*P* = 0.012). Figure 3 also shows that 11 patients with a historical HbA1c-within-target had an elevated SAF >1.28 (mean SAF of controls + 1 SD), whereas 1 patient with a historical HbA1c-above-target had a decreased SAF <1.00 (mean SAF of controls −1 SD).

Univariate linear regression analyses (Table 2) showed a significant impact of age, diabetes duration, current HbA1c, historical HbA1c, current HbA1c (HbA1c-above-target/Hba1c-within-target), and historical HbA1c (HbA1c-above-target/Hba1c-within-target) on SAF. We found no significant impact of gender on SAF. Adjusted R^2^ did not exceed 0.207.

**Table 2:** Univariate and multiple linear regression analyses (*n* = 77) to assess the impact of the following covariables on SAF: age; diabetes duration; gender; current HbA1c (current value, and HbA1c-above-target/ HbA1c-within-target); and historical HbA1c (historical value, and HbA1c-above-target/ HbA1c-within-target)

	Beta coefficient	95 % CI	*P*-value	Adjusted R^2^
Univariate analysis				
Age (years)	0.047	0.026, 0.067	<0.001	0.207
Duration of diabetes (years)	0.025	0.013, 0.038	<0.001	0.172
Sex (male/female)	0.047	−0.056, 0.150	0.366	−0.002
Current HbA1c (%)	0.056	0.019, 0.092	0.003	0.097
Historical HbA1c (%) (*n* = 73)	0.057	0.003, 0.111	0.040	0.045
Current HbA1c: HbA1c-above-target vs. Hba1c-within-target	0.034	−0.104, 0.172	0.628	−0.010
Historical HbA1c: HbA1c-above-target vs. Hba1c-within-target (*n* = 73)	0.028	−0.092, 0.147	0.645	−0.011
Multiple linear regression analysis				
*Model 1 (n = 77)*				0.265
Age (years)	0.040	0.019, 0.061	<0.001	
Diabetes duration 4 to <10 years vs. ≤ 4 years	0.065	−0.044, 0.172	0.239	
Diabetes duration ≥10 years vs. ≤ 4 years	0.187	0.053, 0.320	0.007	
*Model 2 (n = 73)*				0.235
Age (years)	0.043	0.022, 0.065	<0.001	
Diabetes duration 4 to <10 years vs. ≤ 4 years	0.056	−0.064, 0.175	0.356	
Diabetes duration ≥10 years vs. ≤ 4 years	0.179	0.030, 0.328	0.019	
Current HbA1c: HbA1c-above-target vs. Hba1c-within-target	0.012	−0.138, 0.162	0.872	
Historical HbA1c: HbA1c-above-target vs. Hba1c-within-target	−0.048	−0.172, 0.077	0.446	

HbA1c-above-target: > 7.5 %/59 mmol/mol; Hba1c-within-target HbA1c: ≤ 7.5 %/59 mmol/mol

For the multiple linear regression analyses (Table 2), Model 1 showed a significant effect of ‘diabetes duration ≥10 years’ and age on SAF. In Model 2 the addition of the current HbA1c (HbA1c-above-target/Hba1c-within-target) and historical HbA1c (HbA1c-above-target/Hba1c-within-target) showed a significant impact of age and diabetes duration but not of current or historical HbA1c on SAF. The adjusted R^2^ increased to 0.235.

## Discussion

Consistent with previous studies [5, 14, 15], patients with type 1 diabetes showed significantly higher SAF than controls, both for the group as a whole and across all age categories. It is important to note that this was already apparent for the lowest age category (age 11–12 years). SAF appeared to increase faster in the elder adolescents for both patients and controls and with diabetes duration in patients. Differences in SAF between current HbA1c-within-target and HbA1c-above-target patients and between historical Hba1c-within-target and HbA1c-above-target patients were small. In addition, SAF was only weakly associated with diabetes duration and HbA1c (both current and historical) in our homogeneous group of Dutch Caucasians, consistent with SIF findings from Felipe et al. [5]. This association disappeared when adjusting for diabetes duration and age. Interestingly, a subgroup of patients with a Hba1c-within-target had an elevated SAF. Previous studies showed conflicting results on the association between historical HbA1c and SAF [7, 22]. A strong correlation between historical HbA1c and SAF would be expected, as SAF is believed to be at least partly caused by hyperglycemia-induced superoxide and carbonyl damage, resulting in permanent damage to long-lived proteins such as collagen [3, 23]. However, in this study, a strong association between historical HbA1c and SAF could not be demonstrated. An explanation may be that the period during which historical HbA1c was determined was too short or that median intra-individual HbA1c is an inadequate parameter to express historical HbA1c. Alternatively, as sAGEs are considered to be formed by various pathways [7, 23, 24], the influence of hyperglycemia on SAF may also be overestimated. It is intriguing to see that some patients show an elevated SAF despite having a HbA1c-within-target. This may be explained by genetic factors influencing either the level of glycation of HbA1c or by factors that influence AGE formation such as polymorphisms of the AGE-receptor (*RAGE*) gene [25] or the *NAT2* acetylator [26]. Also, oxidative/carbonyl stress may play a role [24, 27], which may be hypoglycemia-related [28]. An outstanding question is if this heterogeneity in patients reflects differences in risk for complications. If so, then SAF measurement in this subgroup may provide information on risk for complications independent of HbA1c. However, one should bear in mind that Sun et al. showed in the Medalist Joslin study group that certain types of plasma AGEs are associated with risk of complications and others are protective [29]. In addition, Conway et al. suggest that also resistance to AGEs may play a role [30].

A strength of this study was that adjustment for skin color was not necessary, as measured patients and controls were from the same ethnic background (Caucasian). Homogeneity of the patient population supports internal validity. However, the results cannot be applied to non-Caucasians and therefore generalizability is lower. Also, we studied the age range 11–19 years in more detail when compared with previous studies [5, 12, 31], showing clearly that children and adolescents with type 1 diabetes in the age category 11–12 years already have elevated SAF. We took into account the use of skin care products, as these can affect SAF readings [32]. One limitation of our study as well as previous studies [5, 15] is that we were unable to quantify measurement errors in terms of coefficient of variation. To reduce measurement error as much as possible, only one type of AGE-reader was used and SAF measurements were performed in triplicate. However, when measuring SAF is to be of use in routine clinical practice, precision CV of these measurements will have to be assessed to be able to distinguish measurement error from clinically meaningful SAF measurements. SAF readings may be confounded by a number of behavioral factors such as dietary factors and fasting state [33]. Additional factors such as smoking and exercise are implicated in the accumulation of SAF [20, 28]. We could not adjust for these factors. BMI may influence SAF, in particular in individuals with central obesity [34]. We did not extend the BMI analyses as only four patients had a BMI >+2 SDS.

## Conclusion

In summary, children and adolescents with type 1 diabetes show higher SAF than controls. Age and duration of diabetes are weakly associated with SAF. In the majority of patients, SAF does not seem to provide information additional to HbA1c. However, in a subgroup of patients with HbA1c-within-target an elevated SAF was observed. For this subgroup, measuring SAF may have added value in identifying patients that are at high risk for complications. Further longitudinal, prospective studies should provide insight into whether SAF measurement in youngsters has predictive value for the development of complications during the disease course of type 1 diabetes and how this is related to glycemic control.

## Abbreviations

AGE: Advanced glycation end products
BMI: Body mass index
DBP: Diastolic blood pressure
HbA1c: Glycated hemoglobin
IQR: Interquartile range
RAGE: Receptor for AGE
SAF: Skin autofluorescence
sAGE: Skin advanced glycation end products
SBP: Systolic blood pressure
SDS: Standard deviation scores
SIF: Skin intrinsic fluorescence
